# Integration of a *Galdieria* plasma membrane sugar transporter enables heterotrophic growth of the obligate photoautotrophic red alga *Cynanidioschyzon merolae*

**DOI:** 10.1002/pld3.134

**Published:** 2019-04-08

**Authors:** Takayuki Fujiwara, Shunsuke Hirooka, Mizuna Mukai, Ryudo Ohbayashi, Yu kanesaki, Satoru Watanabe, Shin‐ya Miyagishima

**Affiliations:** ^1^ Department of Gene Function and Phenomics National Institute of Genetics Mishima Shizuoka Japan; ^2^ JST‐Mirai Program Japan Science and Technology Agency Kawaguchi Saitama Japan; ^3^ Department of Genetics Graduate University for Advanced Studies (SOKENDAI) Mishima Shizuoka Japan; ^4^ Department of Bioscience Tokyo University of Agriculture Tokyo Japan; ^5^ NODAI Genome Research Center Tokyo Japan; ^6^ Research Institute of Green Science and Technology Shizuoka University Shizuoka Japan

**Keywords:** *Cyanidioschyzon merolae*, *Galdieria sulphuraria*, heterotroph, mixotroph, plasma membrane sugar transporter

## Abstract

The unicellular thermoacidophilic red alga *Cyanidioschyzon merolae* is an emerging model organism of photosynthetic eukaryotes. Its relatively simple genome (16.5 Mbp) with very low‐genetic redundancy and its cellular structure possessing one chloroplast, mitochondrion, peroxisome, and other organelles have facilitated studies. In addition, this alga is genetically tractable, and the nuclear and chloroplast genomes can be modified by integration of transgenes via homologous recombination. Recent studies have attempted to clarify the structure and function of the photosystems of this alga. However, it is difficult to obtain photosynthesis‐defective mutants for molecular genetic studies because this organism is an obligate autotroph. To overcome this issue in *C. merolae*, we expressed a plasma membrane sugar transporter, GsSPT1, from *Galdieria sulphuraria*, which is an evolutionary relative of *C. merolae* and capable of heterotrophic growth. The heterologously expressed GsSPT1 localized at the plasma membrane. GsSPT1 enabled *C. merolae* to grow mixotrophically and heterotrophically, in which cells grew in the dark with glucose or in the light with a photosynthetic inhibitor 3‐(3,4‐dichlorophenyl)‐1,1‐dimethylurea (DCMU) and glucose. When the *GsSPT1* transgene multiplied on the *C. merolae* chromosome via the *URA*_*Cm‐Gs*_ selection marker, which can multiply itself and its flanking transgene, GsSPT1 protein level increased and the heterotrophic and mixotrophic growth of the transformant accelerated. We also found that GsSPT1 overexpressing *C. merolae* efficiently formed colonies on solidified medium under light with glucose and DCMU. Thus, GsSPT1 overexpresser will facilitate single colony isolation and analyses of photosynthesis‐deficient mutants produced either by random or site‐directed mutagenesis. In addition, our results yielded evidence supporting that the presence or absence of plasma membrane sugar transporters is a major cause of difference in trophic properties between *C. merolae* and *G. sulphuraria*.

## 1 INTRODUCTION

Photosynthesis by cyanobacteria and photosynthetic eukaryotes generates organic compounds such as carbohydrates and amino acids from inorganic materials by using light energy and introduces these products into ecosystems. Studies on photosynthesis and its relationship with other cellular activities are major targets in plant biology and have been conducted using various organisms. Among photosynthetic organisms, unicellular algae have several advantages compared to terrestrial plants. A unicellular algal culture often provides a homogeneous population in terms of cell types and surrounding environment (including pH, temperature, light strength, and inorganic nutrient condition). This contrasts with terrestrial plants in which many different types of cells and tissues differentiate, with each cell exposed to a different environment. In addition, some unicellular algal species exhibit relatively simpler cellular and genomic architectures among eukaryotes.

Among unicellular algae, molecular genetic studies on photosynthesis and its relationship with other cellular activities have thus far been conducted mainly in the cyanobacterium *Synechocystis* sp. PCC 6803 (Astier, Elmorjani, Meyer, Joset, & Herdman, 1984; Peschek, Obinger, & Renger, 2011) and the green alga *Chlamydomonas reinhardtii* (reviewed by Rochaix, 2004). This is because procedures for genetic modifications have been well established in these algae. In addition, these algae are capable of heterotrophic growth in media supplied with an organic carbon source (56 mM of glucose for *Synechocystis* sp. or 17.5 mM acetate for *C. reinhardtii*). Owing to these characteristics, photosynthesis‐deficient mutants can be analyzed. *C. reinhardtii* has largely contributed to studies on photosynthesis in eukaryotes. The well‐defined sexual cycle of this alga enables dissection of mutations in the nuclear or chloroplast genome based on Mendelian or maternal inheritance, respectively (Levine, 1968). Structural studies and site‐directed mutagenesis of chloroplast DNA have been adopted to analyze the function of respective subunits of photosystems (Rochaix, Fischer, & Hippler, 2000). However, the overexpression of a given gene of interest and gene expression of other organisms from the nuclear genome are very difficult because of silenced transgenes (Cerutti, Johnson, Gillham, & Boynton, 1997). In addition, any reliable procedures for site‐directed mutagenesis in the nuclear genome have not been developed.

The unicellular thermoacidophilic red alga *C. merolae* is an emerging model organism of photosynthetic eukaryotes. The nuclear and organelle genomes have been completely sequenced and the nuclear genome size (16 Mbp) is very small compared to *C. reinhardtii*, 120 Mb, and *Arabidopsis thaliana*, 157 Mb, with little genetic redundancy (Matsuzaki et al., 2004). Its cells do not possess a rigid cell wall and, thus, it is easy to extract cellular contents. Procedures for nuclear (Fujiwara, Ohnuma, Yoshida, Kuroiwa, & Hirano, 2013; Imamura et al., 2010) and chloroplast (Zienkiewicz, Krupnik, Drożak, Golke, & Romanowska, 2017) genome modification by homologous recombination have been established. A transgene is stably expressed without any gene silencing (Fujiwara, Ohnuma, et al., 2013; Imamura et al., 2010). In addition, conditional gene expression and knockdown are also feasible (Fujiwara et al., 2015; Sumiya, Fujiwara, Kobayashi, Misumi, & Miyagishima, 2014). Thus far, this alga has facilitated studies on cell cycle (Fujiwara, Tanaka, Kuroiwa, & Hirano, 2013; Kobayashi et al., 2009; Miyagishima et al., 2014), division and inheritance of organelles (Imoto et al., 2018; Miyagishima et al., 2003; Yagisawa, Nishida, Kuroiwa, Nagata, & Kuroiwa, 2007; Yoshida et al., 2017), and nitrogen and carbohydrate metabolism (Imamura et al., 2009; Pancha et al., 2018). In addition, there have been recent biochemical, spectroscopic, and high‐resolution structural studies of photosystems in this alga (Antoshvili, Caspy, Hippler, & Nelson, 2018; Busch, Nield, & Hippler, 2010; Krupnik et al., 2013; Pham, Janna Olmos, Chernev, Kargul, & Messinger, 2018; Pi et al., 2018).

In terms of photosynthesis, in contrast to green algae and terrestrial plants, red algae and cyanobacteria possess phycobilisomes as light‐harvesting antenna associated with photosystem II (PS II). However, unlike cyanobacteria, red algae possess the light‐harvesting complex (LHC) associated with photosystem I (PS I) as do green algae and land plants (together grouped as Viridiplantae) (Green & Durnford, 1996). Thus, the red algal photosystems are believed to an intermediate between that of cyanobacteria and Viridiplantae. Recent structural and biochemical analyses in *C. merolae* have started to elucidate similarities and differences in the photosystems of red algae, cyanobacteria and Viridiplantae (Antoshvili et al., 2018; Pi et al., 2018) and how photosystems are adapted to thermoacidophillic environments (Krupnik et al., 2013). In this regard, if molecular genetic approaches are integrated into such studies with *C. merolae*, it will give further important insights into the evolution of photosynthesis. In addition, the simple features of this alga will facilitate studies on intra‐organellar interactions based on photosynthesis. However, *C. merolae* is an obligate photoautotroph and thus any photosynthesis‐deficient mutants will be lethal in contrast to *C. reinhardtii*.

Regarding this matter, recent studies showed that *C. merolae* is capable of heterotrophic growth under dark when a very high concentration (more than 400 mM) of glycerol was exogenously supplied, although growth was very slow (Moriyama, Mori, Nagata, & Sato, 2018; Moriyama, Mori, & Sato, 2015). However, this finding raised the possibility that *C. merolae* grows well heterotrophically if the cells efficiently take up an exogenous organic carbon source. In contrast to *C. merolae*, its evolutionary relative *Galdieria sulphuraria* is able to assimilate more than 50 different carbon sources, such as sugars and sugar alcohols (Gross, 1999; Gross & Oesterhelt, 1999; Gross & Schnarrenberger, 1995; Rigano, Aliotta, Rigano, Fuggi, & Vona, 1977; Rigano, Fuggi, Martino, Rigano, & Aliotta, 1976). Thus, it is probably feasible to obtain photosynthesis‐deficient mutants of *G. sulphuraria* by random mutagenesis. However, further molecular genetic analyses will be limited because *G. sulphuraria* is not genetically tractable. In addition, a rigid cell wall of *G. sulphuraria* hampers extraction and fractionation of cellular contents in mild processing conditions. This situation contrasts with *C. merolae* which does not possess a rigid cell wall.

Previous genomic analysis of *G. sulphuraria* identified 28 potential plasma membrane sugar transporters and it was shown that *C. merolae* does not possess any of them (Barbier et al., 2005). Among the transporters, the function of the three proteins (GsSPT1 [*G. sulphuraria* sugar and polyol transporter 1], GsSPT2, and GsSPT4) was characterized by expressing these proteins in a sugar uptake‐deficient yeast mutant (Schilling & Oesterhelt, 2007). The study showed that these three transporters took up monosaccharides and that substrate specificities were broad and largely redundant, except for glucose, which was only taken up by SPT1 (Schilling & Oesterhelt, 2007). In the obligate photoautotrophic diatom *Phaeodactylum tricornutum*, heterologous expression of a human glucose transporter gene *glut1* enabled this alga to grow heterotrophically in the dark with exogenous glucose (Zaslavskaia et al., 2001). Based on this information, we expected that the expression of a *G. sulphuraria* plasma membrane sugar transporter in *C. merolae* would lead to efficient heterotrophic growth. Because *G. sulphuraria* inhabits sulfuric hot springs as does *C. merolae*, its plasma membrane transporters will efficiently function in a thermoacidophilic environment (42°C and pH 2.5 are optimal conditions for *C. merolae* growth).

As a first step to facilitate molecular genetic studies related to photosynthesis in *C. merolae*, in this study, we expressed *G. sulphuraria* GsSPT1 in *C. merolae*. We showed that the heterologously expressed GsSPT1 localizes to the plasma membrane in *C. merolae* and enables *C. merolae* to grow heterotrophically and mixotrophically in the presence of exogenous glucose. We also showed that multi‐copy integration of the Gs*SPT1* transgene into a chromosomal locus leads to overexpression of the protein and accelerates heterotrophic and mixotrophic growth rates. Using these strains and further improvements will be useful for molecular genetic studies on photosynthesis. In addition, the results experimentally suggested that the presence or absence of the plasma membrane sugar transporters is a major cause for the difference in trophic properties between *G. sulphuraria* and *C. merolae*.

## 2 MATERIALS AND METHODS

### 2.1 Algal cultures

The wild‐type *C. merolae* 10D (NIES‐3377) and the *C. merolae* M4‐derived transformants were maintained in inorganic MA2 medium, which was adjusted to pH 2.3 with H_2_SO_4_ (Ohnuma, Yokoyama, Inouye, Sekine, & Tanaka, 2008), in 60 ml tissue culture flasks (90026, TPP) under continuous light (photon flux 50 μmol m^−2^ s^−1^) at 42°C. The uracil‐auxotrophic mutant M4 (a derivative of *C. merolae* 10D, which has a mutation in the *URA* gene; Minoda, Sakagami, Yagisawa, Kuroiwa, & Tanaka, 2004) was maintained in MA2 medium supplemented with uracil (0.5 mg/ml) and 5‐fluoroorotic acid monohydrate (0.8 mg/ml).

The pH of cultures in several different conditions was checked 10 days after inoculation. The pH values were between 2.2 and 2.4. These were within the range for the optimal growth of *C. merolae* (pH 1.0–4.0; Kuroiwa et al., 2017).

### 2.2 Preparation of linear DNA for transformation of *C. merolae*

The primers used in this study are listed in Table S1. Linear DNA for transformation of *C. merolae* M4 was prepared as described below.

To produce the Ap‐*SPT1* strain, we constructed the plasmid pU‐Ap‐*SPT1* as follows. A plasmid vector backbone (pGEMT‐easy; for amplification in *E. coli*), 5ʹ‐upstream sequence of *URA* locus (for homologous recombination with M4 chromosomal DNA), *APCC* promoter, β*‐tubulin* terminator*, URA* marker (for *C. merolae* transformant selection), and its flanking downstream sequence (for homologous recombination with M4 chromosomal DNA) were amplified as linear DNA by PCR with the primer set No. 1/2 and pU‐*APCC*p (Fujiwara et al., 2015) as a template. Additionally, 3× HA‐coding sequence was amplified with the primer set No 3/4 and pBSb‐THA (Ohnuma et al., 2008) as a template. GsSPT1 ORF (Gen Bank ID: EF166070.1) was amplified by PCR with the primer set No. 5/6 and cDNA of *G. sulphuraria* 074G as a template. Then, 3× HA‐coding sequence and Gs*SPT1* ORF were cloned between the *APCC* promoter and β*‐tubulin* terminator of the above DNA amplified from pU‐*APCC*p via the In‐Fusion Cloning Kit (Takara). The resultant plasmid was named pU‐Ap‐*SPT1*. Finally, we amplified the assembled fragment containing the 5ʹ‐upstream sequence of *URA* locus, *APCC* promoter, 3× HA‐coding sequence, Gs*SPT1* ORF, the β*‐tubulin* terminator, *URA* marker, and its flanking downstream sequence by PCR with the primer set No. 7/8 and the pU‐Ap‐*SPT1* as a template for transformation of *C. merolae* M4.

To produce the Cp‐*SPT1* strain, we constructed the plasmid pD‐Cp‐*SPT1 *cm as follows. A plasmid vector backbone (pQE80), a portion of CMD184C locus (for homologous recombination with M4 chromosomal DNA), β*‐tubulin* terminator*, URA*_Cm‐Gs_ marker (for *C. merolae* transformant selection), and 3ʹ‐downstream genomic sequence of CMD184C (for homologous recombination with M4 chromosomal DNA), was amplified as linear DNA by PCR with the primer set No. 9/10 and pD184‐O250‐EGFP‐*URA*_Cm‐Gs_ (Fujiwara, Ohnuma, et al., 2013) as a template. The *CPCC* promoter sequence (500‐bp upstream flanking genomic sequence of *CPCC* ORF) was amplified by PCR with the primer set No. 11/12 and *C. merolae* genomic DNA as a template. Additionally, 3× HA‐coding sequence fused with Gs*SPT1* was codon‐optimized with *C. merolae* codon usage according to the Codon Usage Database in the Kazusa DNA Research Institute (http://awww.kazusa.or.jp/codon/cgi-bin/showcodon.cgi?species=280699) and commercially synthesized. The codon‐optimized sequence of *HA‐SPT1* is described in Material S1. The amplified *CPCC* promoter and the codon‐optimized HA‐*SPT1* were cloned between the CMD184C locus and β*‐tubulin* terminator of the above DNA amplified from pD184‐O250‐EGFP‐*URA*_Cm‐Gs_ via the In‐Fusion Cloning Kit. The resultant plasmid was named pD‐Cp‐*SPT1*. Finally, we amplified the assembled fragment containing a portion of the CMD184C locus, the *CPCC* promoter, HA‐*SPT1*cm, β*‐tubulin* terminator*, URA*_Cm‐Gs_ marker, and 3ʹ‐downstream genomic sequence of CMD184C by PCR with the primer set No. 13/14 and the pD‐Cp‐*SPT1* as a template for transformation of *C. merolae* M4.

To produce the Ap‐*mVENUS* strain, we constructed the plasmid pU‐Ap‐*mVENUS* as follows. The *mVENUS*‐coding ORF, which is codon‐optimized with *C. merolae*, was commercially synthesized (the sequence is described in Material S1). A plasmid vector backbone (pGEMT‐easy), 5ʹ‐upstream sequence of *URA* locus (for homologous recombination with M4 chromosomal DNA), *APCC* promoter, β*‐tubulin* terminator*, URA* marker (for *C. merolae* transformant selection), and its flanking downstream sequence (for homologous recombination with M4 chromosomal DNA) was amplified as a linear DNA by PCR with the primer set No. 1/2 and pU‐*APCC*p (Fujiwara et al., 2015) as a template. Then, the mVENUScm ORF was cloned between *APCC* promoter and β*‐tubulin* terminator of the above DNA amplified from pU‐*APCC*p using the In‐Fusion Cloning Kit. The resultant plasmid was named pU‐Ap‐*mVENUS*. Finally, we amplified the assembled fragment containing the 5ʹ‐upstream sequence of *URA* locus, *APCC* promoter, mVENUS ORF, β*‐tubulin* terminator, the *URA* marker, and its flanking downstream sequence by PCR with the primer sets No. 7/8 and the pU‐Ap‐*mVENUS* as a template for transformation of *C. merolae* M4.

To produce the Cp‐*mVENUS* strain, we replaced the *APCC* promoter of pU‐Cp‐*mVENUS* with *CPCC* promoter as follows. The vector sequence without *APCC* promoter was amplified from pU‐Ap‐*mVENUS* with the primer set No. 15/16. *CPCC* promoter (500‐bp upstream flanking genomic sequence of the *CPCC* ORF) was amplified by PCR with the primer set No. 17/18 and *C. merolae* genomic DNA as a template. The amplified *CPCC* promoter was cloned between the 5ʹ‐upstream sequence of the *URA* locus and mVENUS ORF of the above DNA amplified from pU‐Ap‐*mVENUS* using the In‐Fusion CloningKit. The resultant plasmid was named pU‐Cp‐*mVENUS*. Finally, we amplified the assembled fragment containing the 5ʹ‐upstream sequence of the *URA* locus, *CPCC* promoter, *mVENUS* ORF, β*‐tubulin* terminator, and the *URA* marker by PCR with the primer sets No. 7/8 and the pU‐Cp‐*mVENUS* as a template for transformation of *C. merolae* M4.

After purification via the QIAquick PCR Purification Kit (QIAGEN), 3–5 μg of respective linear construct DNA was used for transformation of *C. merolae* M4. Transformation was performed as previously described (Fujiwara et al., 2015; Kuroiwa et al., 2017).

### 2.3 Measurement of oxygen evolution rates

The wild‐type stock cultures were diluted into 25 ml of fresh MA2 medium to give an OD_750_ of 0.2 in 60 ml tissue culture flasks and then grown for 3 days under continuous light (photon flux 40 μmol m^−2^ s^−1^) at 42°C. Oxygen consumption and evolution rates were measured by an oxygen electrode (Oxytherm system composed of a S1/MINI Clark type electrode disc and OXYT1 electrode control unit controlled by O_2_ view software; Hansatech, Norfolk, England) using ~2 ml of culture. The rates in *C. merolae* were measured at 40°C. The light intensity for the measurement of oxygen evolution by photosynthesis was 100 μE m^−2^ s^−1^. The chlorophyll *a* concentration was determined using the method described by Porra, Thompson, and Kriedemann (1989).

### 2.4 Quantitative PCR

The genomic DNA of *C. merolae* wild‐type, Ap‐*SPT1,* and Cp‐*SPT1* was extracted as previously described (Fujiwara, Ohnuma, et al., 2013). The wild‐type DNA was serially diluted to concentrations of 10, 2.5, 0.63, 0.16, and 0.04 ng/μl in distilled water as templates to construct a standard curve. To measure the copy number of respective genomic regions, wild‐type, Ap‐*SPT1*, and Cp‐*SPT1* genomic DNA was adjusted to 0.5 ng/μl. Quantitative PCR was performed with the StepOnePlus Real‐Time PCR System (Applied Biosystems) with a 20‐μl reaction mixture (10 μl of PowerUp SYBR Green Master Mix [Thermo Fisher Scientific], 0.1 μl of 50 μM forward and reverse primers, 4 μl of each 0.5 ng/μl genomic DNA solution, and 15.8 μl of water). The primers used in this study are listed in Table S1.

### 2.5 Immunoblotting

Cells were harvested by centrifugation at 3,000 *g* for 10 min at 4°C. The cell pellets were lysed with the sample buffer (2% SDS, 62 mM Tris‐HCl pH 6.8, 100 mM DTT, 10% glycerol, and 0.01% BPP) and then sonicated (Cosmobio, Bioruptor UCW‐310, 310 W, 16 cycles of 30 s with a 30 s rest period). Three micrograms of total cellular protein was separated by SDS‐PAGE in each lane on 10% SDS‐polyacrylamide gels and transferred onto polyvinylidene difluoride (PVDF) membranes (Immobilon; Millipore).

The membrane blocking, antibody reactions, and signal detection were performed as previously described (Fujiwara et al., 2015). The anti‐HA antibody (clone 16B12, BioLegend; at a dilution of 1:3,000) was used to detect HA‐SPT1 protein. The anti‐GFP antibody (ab6556, abcam; at a concentration of 0.25 μg/ml) was used to detect mVENUS protein.

### 2.6 Measurement of cellular growth

To examine the growth of wild‐type, Ap‐*SPT1*, and Cp‐*SPT1* strains, respective stock cultures were diluted with fresh MA2 medium to give an OD_750_ of 0.2 and then grown for a week to prepare photoautotrophically grown cells in the same culturing condition.

Then, the cells were inoculated into 16 ml of fresh MA2 medium to give an OD_750_ of 0.1 in 25 cm^2^ tissue culture flasks with a filter cap (90026, TPP). DCMU and glucose were added to the culture to give final concentrations of 10 μM and 25 mM, respectively. The cultures in the flasks were incubated at 42°C with agitation at 120 rpm under continuous light (40 μmol m^−2^ s^−1^) or dark.

### 2.7 Colony formation on a solidified medium

The gellan gum‐solidified medium with starch beds was prepared as previously described (Fujiwara, Ohnuma, et al., 2013; Kuroiwa et al., 2017). Wild‐type, Ap‐*SPT1*, and Cp‐*SPT1* cells were suspended in MA2 liquid medium supplemented with 25 mM glucose alone or 25 mM glucose and 10 μM DCMU. Eight microliters of cellular suspension was inoculated onto a starch bed on a gellan gum‐solidified medium. The starch slurry and the solidified MA2 medium also contained 25 mM of glucose alone or 25 mM glucose and 10 μM DCMU.

Then, solidified medium was incubated in a CO_2_ incubator with 2% of CO_2_ at 42°C under continuous light (40 μmol m^−2^ s^−1^) or dark.

## 3 RESULTS

### 3.1 Expression of the GsSPT1 protein enables *C. merolae* to assimilate exogenous glucose and grow mixotrophically and heterotrophically

**Figure 1:**
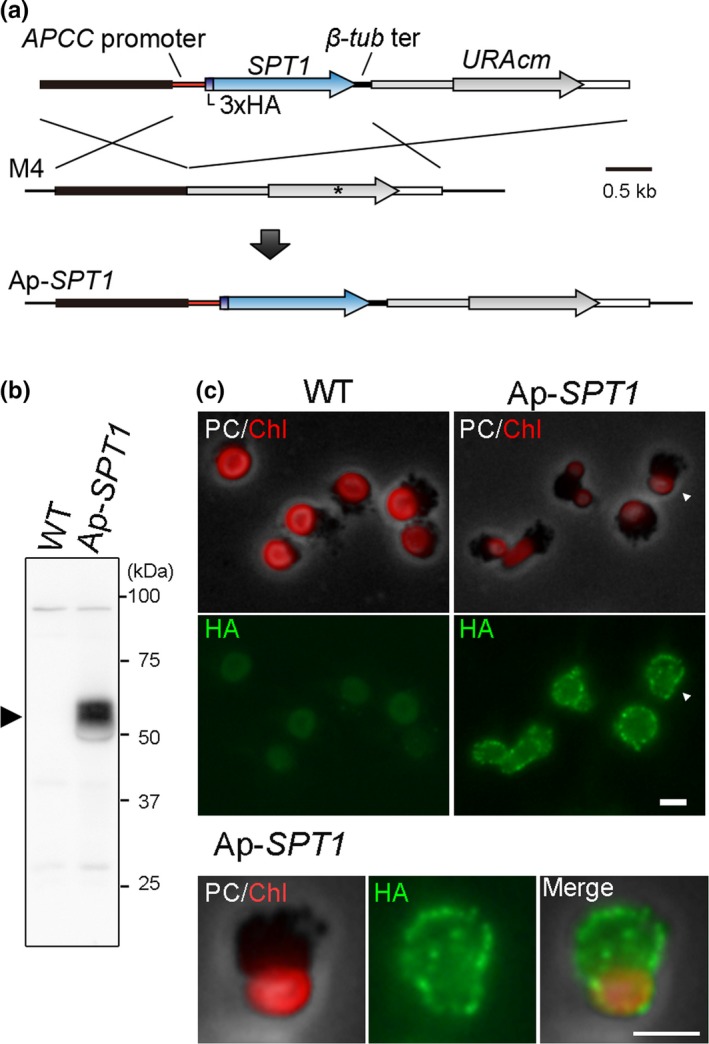
Production of a *Cyanidioschyzon merolae* strain expressing HA‐GsSPT1 (Ap‐*SPT1* strain). (a) A schematic diagram of the insertion of *HA‐SPT1* transgene and *URA* selection marker into the chromosomal *URA* locus. The first line indicates introduced linear DNA, the second line indicates the genomic structure of the uracil‐auxotrophic mutant M4 (derived from *C. merolae* 10D), and the third line indicates genomic structure of Ap‐*SPT1*. The asterisk shows the position of a frameshift mutation in the *URA* gene resulting in truncation of the C‐terminal half of the URA protein in M4. To express HA‐*GsSPT1*,*APCC* promoter (600‐bp upstream flanking sequence of the *APCC* ORF) and β*‐tubulin* terminator (200‐bp downstream flanking sequence of β*‐tubulin* ORF) were connected to the upstream and downstream regions of the *HA‐SPT1* ORF, respectively. (b) Immunoblotting of total cell lysate with an anti‐HA antibody showing expression of HA‐SPT1 in the Ap‐*SPT1* strain. The wild‐type (WT) strain was used as a negative control. The arrowhead indicates the HA‐SPT1 protein that was expressed specifically in Ap‐*SPT1*. (c) Phase‐contrast (PC) and immunofluorescent images of the WT and Ap‐SPT1 cells stained with an anti‐HA antibody. The cell indicated with the arrowheads was enlarged and shown in the images. The red color is autofluorescence of the chloroplast (Chl). Green fluorescence is the signal detecting HA‐SPT1 (HA). Bars = 2 μm

In order to express GsSPT1 protein of *G. sulphuraria* in C. *merolae*, we introduced *GsSPT1* ORF and *C. merolae URA* gene (marker gene for transformant selection) into *URA* (*URA5.3*/CML043C) locus of *C. merolae* M4, an uracil‐auxotrophic derivative of *C. merolae* 10D with a point mutation in *URA* (Figure 1a). In order to detect the protein expression, a sequence encoding 3× HA epitope tag was added just after the start codon of *GsSPT1* ORF. The *HA‐GsSPT1* ORF (*HA‐SPT1*) was designed to be driven by the *APCC*/CMO250C promoter, which has relatively strong constitutive transcriptional activity (Watanabe, Ohnuma, Sato, Yoshikawa, & Tanaka, 2011). As a polyadenylation signal sequence, β*‐tubulin* terminator (200‐bp 3′‐flanking sequence of β*‐tubulin*/CMN263C ORF; Fujiwara, Ohnuma, et al., 2013) was added just after the stop codon. Then the *HA‐SPT1* transgene and the *URA* selection marker were inserted to the mutated *URA* locus of *C. merolae* M4 by homologous recombination. The resultant transformant was named Ap‐*SPT1* (Figure 1a).

HA‐SPT1 expression in the Ap‐*SPT1* strain was confirmed by immunoblotting, using an anti‐HA antibody (Figure 1b). The anti‐HA antibody detected a ~60 kDa band in the Ap‐*SPT1* strain but not in the wild‐type strain (Figure 1b). The size roughly corresponded to the deduced molecular weight of HA‐SPT1 (67 kDa). In addition, immunofluorescence staining with the anti‐HA antibody showed that the HA‐SPT1 protein localized around the cell periphery in Ap‐*SPT1* cells (Figure 1c). Given that SPT1 possesses 12 predicted membrane‐spanning domains (Schilling & Oesterhelt, 2007), this result suggested that HA‐SPT1 localizes at the plasma membrane in Ap‐*SPT1* cells.

**Figure 2:**
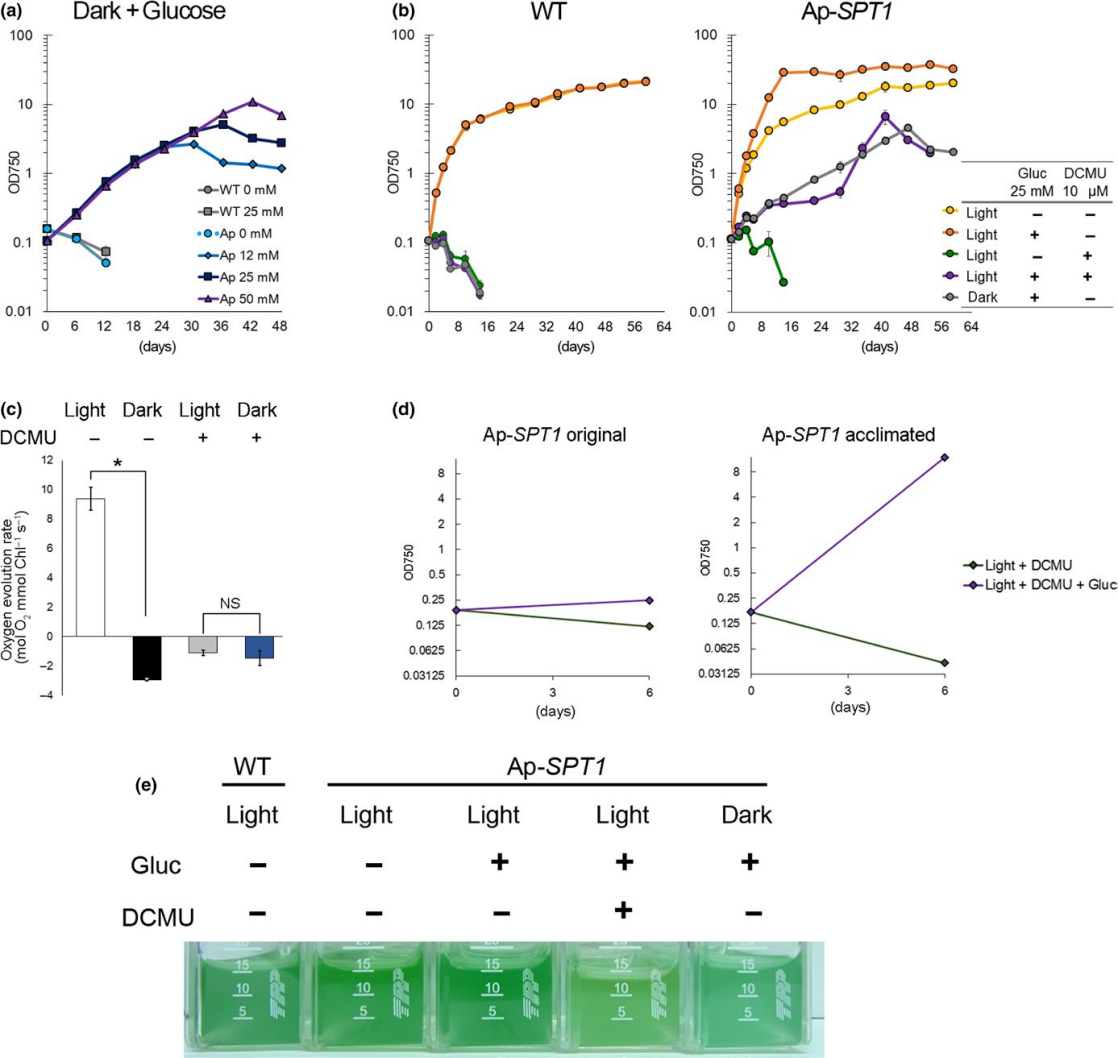
Properties of heterotrophic and mixotrophic growth of Ap‐*SPT1* strain. (a) Growth curves of Ap‐*SPT1* in the inorganic MA2 medium supplemented with 0, 12, 25, and 50 mM glucose in the dark. As a control, the wild‐type (WT) was also grown in MA2 with 0 and 25 mM glucose in the dark. Cultures in the dark were started by inoculating cells maintained photoautotrophically (in MA2 in the light) into respective media. Error bars indicate the standard deviations of three independent cultures. (b) Growth curves of WT and Ap‐*SPT1* in the light (40 μmol m^−2^ s^−1^) or dark with or without 25 mM glucose and/or 10 μM DCMU. Error bars indicate the standard deviations of three independent cultures. (c) Oxygen evolution rates of WT in the light or dark with or without DCMU. Error bars indicate the standard deviations of three independent cultures. *p <* *0.05; NS, not significant (*t*‐test). (d) Growth of Ap‐*SPT1* in the light or dark with or without DCMU in the presence of glucose. The left graph shows growth of Ap‐*SPT1* when the cells grown autotrophically were inoculated at day 0. The right graph shows growth of Ap‐*SPT1* when the cells in the light with glucose and DCMU (day 35 in b) were inoculated at day 0. (e) Image showing the WT and Ap‐*SPT1* cultures grown under respective conditions (at day 13 in b)

To examine whether SPT1 expression enables *C. merolae* cells to grow heterotrophically by taking up exogenous glucose, Ap‐*SPT1* cells were cultured in inorganic MA2 medium supplemented with 0, 12, 25, and 50 mM glucose in the light and dark, and the growth rate was compared with that of the wild‐type (Figure 2a). Whereas the wild‐type did not grow in the dark, regardless of the presence or absence of exogenous glucose, the Ap‐*SPT1* strain grew in the dark in the presence of glucose (Figure 2a). Thus, Ap‐*SPT1* cells assimilated exogenous glucose and were able to grow heterotrophically. The growth rate of the Ap‐*SPT1* strain was almost constant in medium supplemented with different glucose concentrations (12, 25, or 50 mM glucose; Figure 2a). However, the duration of growing phase was longer, and the final cellular concentration was higher when a higher concentration of exogenous glucose was supplied (Figure 2a). The similar initial growth rate among different concentrations of exogenous glucose was probably because of limitation of glucose uptake activity of the HA‐SPT1 proteins. In addition, the difference in their final cellular concentrations was probably because of exhaustion of exogenous glucose.

We compared the growth of Ap‐*SPT1* under autotrophic, mixotrophic, and heterotrophic conditions. The wild‐type strain grew at almost the same rate in the light (40 μmol m^−2^ s^−1^) regardless of the presence or absence of exogenous glucose (25 mM glucose was added to the medium at the onset of culture; Figure 2b). In contrast to the wild‐type, in the light, the Ap‐*SPT1* strain grew faster in the presence of exogenous glucose than in the absence of glucose (Figure 2b). In addition, in the presence of exogenous glucose, Ap‐*SPT1* cells grew faster in the light than the dark (Figure 2b). These results indicated that Ap‐*SPT1* cells are capable of mixotrophic as well as heterotrophic growth.

We next examined the effect of photosynthetic inhibition on Ap‐*SPT1* growth upon the addition of DCMU into culture. We first confirmed that the addition of 10 μM DCMU completely abolishes photosynthetic oxygen evolution in *C. merolae* cultured under photoautotrophic condition (in an inorganic medium at 40 μmol m^−2^ s^−1^) as follows. In contrast to the culture without DCMU, the oxygen evolution at 100 μmol m^−2^ s^−1^ became almost the same as that in the dark (the oxygen consumption is because of respiration) (Figure 2c). The results showed that 10 μM DCMU completely blocked linear electron flow in the photosystems.

The addition of DCMU completely abolished the growth of the wild‐type in the light, both in the absence and presence of exogenous glucose (Figure 2b). In contrast to the wild‐type, Ap‐*SPT1* cells grew in the presence of DCUM in the light when exogenous glucose was added to the culture (Figure 2b). However, the growth of Ap‐*SPT1* cells suddenly accelerated from day 29 until its cell concentration reached OD_750_ = ~6.5 at day 41 (Figure 2b; completely independent culture performed on a different day reproduced a similar result [Figure S1a]). This sudden acceleration of growth was probably not caused by acquisition of DCMU resistance by the photosystem or degradation of DCMU based on following results. We harvested a portion of Ap‐*SPT1* culture in the light with glucose and DCMU at day 35 (during the accelerated growth; named “acclimated cells”), harvested cells, and then cell pellets were inoculated into fresh MA medium with DCMU and with or without glucose in the light.

The acclimated cells continued to grow in the presence of glucose with DCMU but did not in the absence of glucose with DCMU (Figure 2d), indicating that DCMU still abolished phototrophic growth of acclimated Ap‐*SPT1* cells. Acclimated Ap‐*SPT1* cells grew faster than the original Ap‐*SPT1* cells (which were cultured photoautotrophically in MA2 in the light) in fresh medium with DCMU and glucose in the light (Figure 2d; completely independent culture performed on a different day reproduced a similar result [Figure S1b]). These results suggested that Ap‐*SPT1* was acclimated to culture conditions (DCMU and glucose in the light).

Ap‐*SPT1* cells grown with glucose and DCMU in the light and with glucose in the dark became paler and bluer, respectively, than the wild‐type and Ap‐*SPT1* cells grown photoautotrophically (Figure 2e). Thus, Ap‐*SPT1* cells likely changed composition of their photosynthetic pigments to adjust their metabolic states to non‐photosynthetic growth.

### 3.2 Increased GsSPT1 levels via the *CPCC* promoter and multi‐copy integration of the *GsSPT1* transgene with the *URA*_*Cm‐Gs*_ selection marker

As described above, we succeeded in producing a *C. merolae* strain capable of heterotrophic growth. However, heterotrophic growth of Ap‐*SPT1* was slower compared to photoautotrophic growth of *C. merolae*. To improve the heterotrophic growth rate, we attempted to enhance the rate of glucose uptake by increasing cellular HA‐SPT1 protein levels. To this end, we employed the following strategies: (1) codon optimization of *HA‐SPT1*, (2) use of a stronger promoter, and (3) multi‐copy integration of *HA‐SPT1* transgene into the chromosome.

Regarding (1), we optimized all codons of *HA‐SPT1* according to *C. merolae* codon usage, referring to the codon usage database in the Kazusa DNA Research Institute.

**Figure 3:**
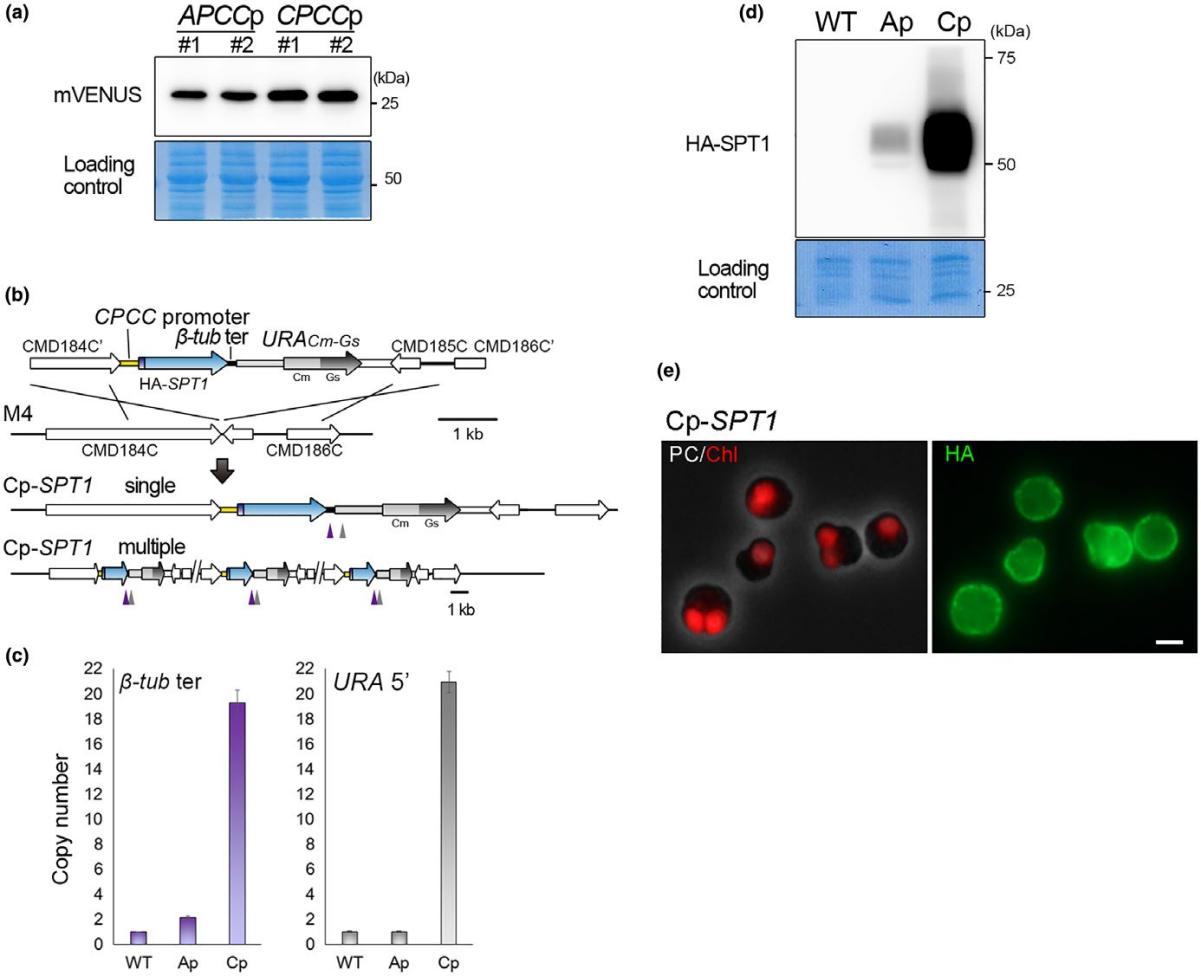
Production of a *Cyanidioschyzon merolae* strain overexpressing HA‐GsSPT1 (Cp‐*SPT1* strain) by multi‐copy integration of the transgene. (a) Immunoblotting with an anti‐GFP antibody to compare activities of *APCC* and *CPCC* promoters. The CBB‐stained PVDF membrane is shown as a loading control. GFP was expressed by *APCC* promoter (*APCC*p; 600‐bp upstream flanking sequence of the *APCC* ORF) or *CPCC* promoter (*CPCC*p; 500‐bp upstream flanking sequence of the *CPCC* ORF) in *C. merolae*. Two independent transformed clones (#1 and #2) for *APCC*p and *CPCC*p were analyzed, respectively. (b) A schematic diagram of the insertion of the *HA‐SPT1* transgene and *URA*_*C*__*m‐Gs*_ selection marker into the chromosomal region between CMD184C and CMD185C loci. The first line indicates the introduced linear DNA, and the second line indicates the genomic structure of *C. merolae* M4. The third and fourth lines indicate the genomic structure of the Cp‐*SPT1* strain in which a single‐copy and multi‐copy of the *HA‐SPT1* and *URA*_*C*__*m‐Gs*_ is integrated into the chromosome, respectively. To express HA‐*GsSPT1*,*CPCC* promoter and β*‐tubulin* terminator were connected to the upstream and downstream regions of the *HA‐* SPT1 ORF (codon‐optimized to *C. merolae*), respectively. (c) Quantitative PCR analyses showing the copy number of transgenes in Ap‐*SPT1* and Cp‐*SPT1* strains. The wild‐type (WT) strain was used as a control. The positions of the amplified sequences were indicated in b (purple arrowhead, β*‐tubulin* terminator; gray arrowhead; *URA* promoter). The values of purple and gray were normalized against values of a chromosomal region outside the introduced DNA. The primers used in the qPCR assay are shown in Supporting Information Table S1. The sequences which correspond to purple and gray arrowheads and the region outside the introduced DNA were amplified using the primer set No. 19/20, 21/22, and 23/24. The wild‐type genome possesses one copy of the endogenous β*‐tubulin* terminator and *URA* promoter. Besides these endogenous sequences, Ap‐*SPT1* genome possesses another copy of β*‐tubulin* terminator of the transgene (total two copies) but one copy of *URA* promoter because the transgene with *URA* marker was integrated into chromosomal *URA* locus (Figure 1a). Besides the endogenous sequences, Cp‐*SPT1* genomes possess additional (~20) copies of β*‐tubulin* terminator and *URA* promoter of the transgene. Error bars indicate the standard deviations of three technical replicates. (d) Immunoblotting of total cell lysate with an anti‐HA antibody showing expression of HA‐SPT1 in Cp‐*SPT1*. The wild‐type (WT) and Ap‐*SPT1* strains were used as controls. The CBB‐stained PVDF membrane is shown as a loading control. (e) Phase‐contrast (PC) and immunofluorescent images of the WT and Cp‐*SPT1* cells stained with an anti‐HA antibody. The red color is autofluorescence of the chloroplast (Chl). Green fluorescence is the signal detecting HA‐SPT1 (HA). Bar = 2 μm

Regarding (2), we tested the *CPCC* promoter based on following reasons. In previous analysis on expressed sequencing tags (ESTs) in *C. merolae*,* CPCC* mRNA was more abundant than that of *APCC* (Matsuzaki et al., 2004). Both APCC and CPCC proteins are components of phycobilisomes, which are light‐harvesting antennae of PS II and are abundant in the chloroplast (Singh, Sonani, Rastogi, & Madamwar, 2015). When the mVENUS protein was expressed by *APCC* and *CPCC* promoters in *C. merolae* stable transformants, the mVENUS protein level was slightly higher in stains in which the transcription was driven by the *CPCC* than the *APCC* promoter (Figure 3a). Thus, we decided to use the *CPCC* promoter to express *HA‐SPT1*.

Regarding (3), we tested the *URA*_*Cm‐Gs*_ selection marker (Imamura et al., 2010). The *C. merolae* URA protein (CMK046C) is composed of the N‐terminal orotidine‐5ʹ‐phosphoribosyl transferase (OPRTase) domain and the C‐terminal orotidine 5ʹ‐monophosphate (OMP)‐decarboxylase domain. In the *URA*_*Cm‐Gs*_ marker, the sequence encoding the C‐terminal OMP‐decarboxylase domain of *C. merolae* is replaced with that of *G. sulphuraria* (Imamura et al., 2010). In our previous study, we integrated the *EGFP* transgene into the same chromosomal locus (intergenic region between CMD184C and CMD185C) together with the *URA* or *URA*_*Cm‐Gs*_ marker (Fujiwara, Ohnuma, et al., 2013). Selection of transformants by *URA* marker resulted in efficient single‐copy insertion of the transgene. In contrast, selection of transformants by the *URA*_*Cm‐Gs*_ marker resulted in multi‐copy insertion of the transgene at high frequencies, accompanied by recombination events at the targeted loci (Fujiwara, Ohnuma, et al., 2013). The number of copies that are integrated into a chromosome is determined by chance and varies among transformed clones (Fujiwara, Ohnuma, et al., 2013). Multi‐copy insertion of the *EGFP* gene resulted in higher accumulation of the EGFP protein in the cytosol than the single‐copy insertion (Fujiwara, Ohnuma, et al., 2013). However, we did not determine whether multi‐copy insertion also occurred for transgenes other than *EGFP*. It was unclear whether the difference in the copy number of integrated transgenes was solely due to differences in the *URA* sequence or its specific combination with the *EGFP* sequence.

We integrated *HA‐SPT1* and *URA*_*Cm‐Gs*_ into the intergenic region between CMD184C and CMD185C and obtained the transformant Cp‐*SPT1* strain (Figure 3b). Quantitative PCR showed that the copy number of the *HA‐SPT1* transgene was a single copy in the Ap‐*SPT1* (*HA‐SPT1* was integrated with the *URA* marker) genome, whereas ~20 copies was present in the Cp‐*SPT1* (*HA‐SPT1* was integrated with the *URA*_*Cm‐Gs*_ marker) genome (Figure 3c). To note, the *SPT1* nucleotide sequence was codon‐optimized in the Cp‐*SPT1* strain and thus different from that of the Ap‐*SPT1* and *G. sulphuraria* 074G strains. Thus, we examined the transgene copy number by using primers corresponding to the 3ʹ‐downstream sequences flanking the *SPT1* ORF. Immunoblotting showed that HA‐SPT1 protein levels were much higher in the Cp‐*SPT1* strain than the Ap‐*SPT1* strain (Figure 3d). Immunofluorescence staining showed that the HA‐SPT1 protein predominantly localized on the cell periphery (Figure 3e). These results showed that HA‐SPT1 protein levels increased without mislocalization of the protein due to increased gene copy number. In addition, the results suggested that the use of the *URA*_*Cm‐Gs*_ marker led to multi‐copy integration of any transgenes and higher expression of the gene products. It should be noted that the *HA‐SPT1* transgene was integrated into different chromosomal loci in the AP‐*SPT1* and CP‐*SPT1* strains (*URA* locus in Ap‐*SPT1*; the intergenic region between CMD184C and CMD185C in Cp‐*SPT1*). We previously showed that the choice of these two sites hardly affected transgene expression levels (Fujiwara et al., 2015).

### 3.3 Increased GsSPT1 levels accelerate *C. merolae* heterotrophic and mixotrophic growth

**Figure 4:**
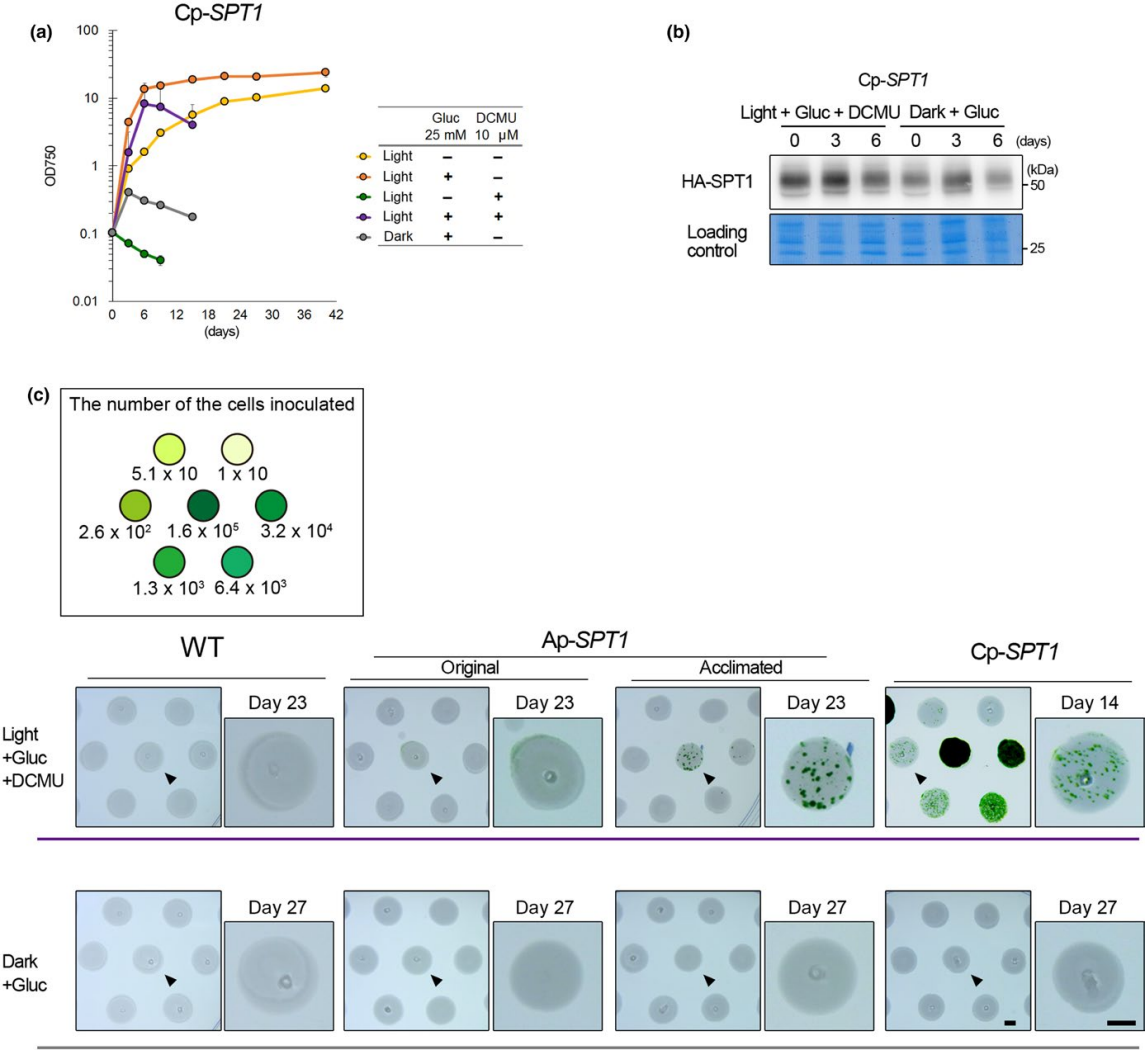
Heterotrophic and mixotrophic growth properties of Cp‐*SPT1* strain. (a) Growth curves of Cp‐*SPT1* in the light (40 μmol m^−2^ s^−1^) or dark with or without 25 mM glucose and/or 10 μM DCMU. Error bars indicate the standard deviations of three independent cultures. (b) Immunoblotting of total cell lysate with an anti‐HA antibody showing expression of HA‐SPT1 in Cp‐*SPT1* in the light with 25 mM glucose and 10 μM DCMU or in the dark with 25 mM glucose. The CBB‐stained PVDF membrane is shown as a loading control. (c) Colony formation of Ap‐*SPT1* and *Cp‐SPT1* cells on the starch bed (white) on the gellan gum‐solidified medium. The wild‐type (WT) strain was used as a control. WT, the original Ap‐*SPT1* and Cp‐*SPT1* cells (grown photoautotrophically in MA2 in the light;) and the acclimated Ap‐*SPT1* cells (grown in MA2 with glucose and DCMU in the light) were inoculated onto the starch bed (~5 mm in diameter) on solidified MA2 medium with 10 μM DCMU and 25 mM glucose in the light (40 μmol m^−2^ s^−1^) or with glucose in the dark. 1.6 × 10^6^, 3.2 × 10^4^, 6.4 × 10^3^, 1.3 × 10^3^, 2.6 × 10^2^, 5.1 × 10, and 1 × 10 cells of each strain were inoculated onto starch beds as shown in the schema. The starch beds indicated with the arrowheads were enlarged and shown on the right of each original image. The number above enlarged starch bed images indicates days after inoculation of cells when the photographs were taken. Bar = 2 mm

We then examined growth patterns of Cp‐*SPT1* cells under various conditions and compared the results with that of Ap‐*SPT1* cells (Figure 4a). As with Ap‐*SPT1* cells (Figure 2b), Cp‐*SPT1* cells grew faster in mixotrophic (in the light with glucose) than in photoautotrophic (in the light without glucose) and heterotrophic conditions (in the dark with glucose) (Figure 4a). Cp‐*SPT1* cells (Figure 4a) grew faster than *AP‐SPT1* cells (Figure 2b) in the presence of glucose probably because of faster uptake of exogenous glucose via higher SPT1 protein levels in Cp‐*SPT1* cells. Under mixotrophic conditions, Ap‐*SPT1* and Cp‐*SPT1* cells reached to OD_750_ = 20 at day 6 and day 10, respectively. Under heterotrophic conditions, Cp‐*SPT1* (Figure 4a) and Ap‐*SPT1* cells (Figure 2b) reached to OD_750_ = 0.4 at day 3 and day 10, respectively. In addition, Cp‐*SPT1* cells (Figure 4a) grew faster in the light with DCMU and glucose than Ap‐*SPT1* cells (Figure 2b).

Although the reason is unclear at this point, the maximum cellular concentration, after which cells started to die, was lower in Cp*‐SPT1* (Figure 4a) cells than Ap*‐SPT1* cells (Figure 2b) in the presence of glucose (in the dark and light with or without DCMU) in contrast to faster growth of Cp*‐SPT1* cells than Ap*‐SPT1* cells in the presence of glucose. In addition, while Ap*‐SPT1* cells grew slower in the light with DCMU glucose (before acclimation) than in the dark (Figure 2b), Cp*‐SPT1* cells grew faster in the light with DCMU glucose than in the dark (Figure 4a).

A previous study showed that *CPCC* mRNA level in the dark was lower than that in the light under photoautotrophic condition (Kawase, Imamura, & Tanaka, 2017). Taking this result into account, we compared the HA‐SPT1 protein level between Cp‐*SPT1* in the dark with glucose and that in the light with glucose and DCMU (Figure 4b). HA‐SPT1 level was not much different between the two conditions (Figure 4b), suggesting that the difference in cellular growth rate between the culture in the dark with glucose and that in the light with glucose and DCMU was not because of difference in *CPCC* promoter activity between the two culture conditions.

Finally, we investigated whether Ap*‐SPT1* and Cp*‐SPT1* cells form colonies on solidified medium under conditions in which photosynthesis is inhibited. This is because production of photosynthesis‐deficient mutants (either by random mutagenesis or designed genetic modification) in *C. merolae* for further analyses requires clone growth, selection, and isolation. To this end, we inoculated the wild‐type, original Ap‐*SPT1*, and Cp‐*SPT1* cells (maintained photoautotrophically in MA2 medium in the light) and acclimated Ap‐*SPT1* (maintained in MA2 medium supplied with DCMU and glucose in the light) onto a gellan gun‐solidified MA2 medium with glucose in the dark or in the light with DCMU. None of the strainformed colonies in the dark with glucose (Figure 4c). In contrast, Ap‐*SPT1* and Cp‐*SPT1* cells successfully formed colonies in the light with glucose and DCMU (Figure 4c). The visible colony formation was evident for the acclimated *Ap‐SPT1* but not for the original *Ap‐SPT1*. The visible colony formation of the Cp‐*SPT1* cells was more efficient and took a shorter period of time than Ap‐*SPT1* cells (no colony after 23 days for the original Ap‐*SPT1*, >30 colonies for the acclimated Ap‐*SPT1* after 23 days, and >50 colonies for Cp‐*SPT1* after 14 days; Figure 4c).

## 4 DISCUSSION

### 4.1 Establishment and property of heterotrophic *C. merolae* cells via the SPT protein

The expression of a *G. sulphuraria* plasma membrane sugar transporter GsSPT1 enabled obligate photoautotrophic *C. merolae* to grow heterotrophically and mixotrophically by taking up glucose from the medium. The results experimentally suggested that the presence or absence of plasma membrane sugar transporter distinguishes trophic properties between *C. merolae*, an obligate photoautotroph, and *G. sulphuraria*, which grows mixotrophically and heterotrophically as well as photoautotrophically.

The transformants successfully formed colonies in the light with exogenous glucose when photosynthesis was inhibited with DCMU. These transformants or further modified strains, in certain cases combining with already developed inducible gene expression/suppression systems, will facilitate analysis of photosynthesis‐deficient mutants produced either by random or site‐directed mutagenesis.

In addition, strains capable of heterotrophic/mixotrophic growth will enable us to compare heterotrophically, mixotrophically, and autotrophically growing cells. This comparison will be useful for studying relationships of photosynthetic activities in the chloroplast to metabolism in other cellular compartments, cell cycle progression, nuclear gene expression, and circadian rhythms. These kinds of studies have been technically hampered in obligate photoautotrophic *C. merolae* because cellular growth ceases when photosynthesis does not occur. During this study, we also showed that the transformant selection with *URA*_*Cm‐Gs*_ marker resulted in multi‐copy insertion of transgenes in contrast to selection with the *URA* marker, which integrates only a single copy. In addition, we showed that the multi‐copy insertion of the transgene lead to higher protein expression than the single‐copy insertion. The reason why *URA*_*Cm‐Gs*_ marker with its flanking transgenes is multiplied in the genome is unclear at this point. The chimeric URA_Cm‐Gs_ enzyme likely possesses less activity than the native URA enzyme in *C. merolae* cells. Thus, for transformant selection on a medium without uracil, only colonies with multiple *URA*_*Cm‐Gs*_ markers with transgenes, in which higher expression of the URA_Cm‐Gs_ protein compensated its lower activity, would be selected. Consistent with this assumption, we usually got a smaller number of transformant colonies under *URA*_*Cm‐Gs*_ selection than *URA* selection.

Although we have succeeded in producing heterotrophic/mixotrophic *C. merolae* strains, we also have obtained results that were not expected before experiments as follows. (1) *SPT1‐*over expressing CP‐*SPT1* strain grew faster and to a higher concentration in the light in the presence of glucose with inhibition of photosynthesis with DCMU than in the dark in the presence of glucose (Figure 4a). (2) In both Ap‐*SPT1* and CP‐*SPT1*, cells started to die under heterotrophic conditions (in the dark with glucose or in the light with glucose and DCMU) immediately after they reached a maximum concentration (Figure 2a, b; Figure 4a). This contrast with autotrophic and mixotrophic conditions and also wild‐type in autotrophic condition in which cells continued to live even after the cells reached the maximum concentration (Figure 2a, b; Figure 4a). (3) Increase in GsSPT1 level (Cp‐*SPT1* compared to Ap‐*SPT1* strain) accelerated heterotrophic and mixotrophic growth rates (Figure 2b; Figure 4a). However, the increase in GsSPT1 level decreased the maximum cellular concentration, after which cells started to die, in the dark with glucose (Figure 2b; Figure 4a).

Regarding (1), our results suggested that light is somehow required for efficient heterotrophic growth of Cp‐*SPT1*, even though photosynthetic carbon fixation is blocked with DCMU. One possibility is that light is required as a signal for cellular growth. Another possibility is that cyclic electron flow around PS I producing ATP is required for efficient heterotrophic growth. DCMU blocks electron flow from PS II to plastoquinone, shutting down the linear electron flow through PS II and PS I and blocking the reduction of NADP+ to NADPH and carbon fixation. However, DCMU does not inhibit cyclic electron flow around PS I, which leads to ATP production. In this regard, cyclic electron flow can be suppressed by DBMIB, but it exhibits high toxicity in *C. merolae*. Thus, it was not feasible to examine the effect of cyclic electron flow on cellular growth in this study.

Regarding (2), we determined that photosynthesis is required for cells to survive without growth after getting a maximum cellular concentration in culture. Even without growth, cells will require ATP synthesis to maintain homeostasis. In addition, cyanidialean red algae (*C. merolae*,* Cyanidium* spp., and *Galdieria* spp.) inhabit acidic environments, and a previous study of *Cyanidium caldarium* suggested that ATP is consumed by the plasma membrane ATPase to pump out H+ and prevents acidification of the cytosol (Enami & Kura‐Hotta, 1984).

Regarding (3), with lower levels of GsSPT1 (Ap‐*SPT1*), cultures maintained in the dark with glucose and that in the light with glucose and DCMU reached to similar maximum cellular concentrations. In contrast, with higher levels of GsSPT1 (Cp‐*SPT1*), the maximum concentration increased in culture maintained in the light with glucose and DCMU but decreased in culture maintained in the dark with glucose compared to lower levels of GsSPT1 (Ap‐*SPT1*). Although the reason for these observations is unclear at this point, in the absence of the light stimulus and/or the cyclic electron flow, the rapid glucose inflow likely causes a toxic imbalance between metabolic pathways.

Regarding the physiological changes resulting from direct sugar uptake in GsSPT1‐expressing cells and the mechanisms regulating a metabolic balance between photosynthetic carbon fixation and glucose flux, further studies are required. Hexokinases are known as evolutionarily conserved sugar sensors in eukaryotes and regulate expressions of genes involved in sugar uptake, carbon metabolism and photosynthesis (Aguilera‐Alvarado & Sánchez‐Nieto, 2017; Rolland, Baena‐Gonzalez, & Sheen, 2006). In *G. sulphuraria*, it is assumed that hexokinases are involved in selection of a preferred sugar to be taken up when various kinds of sugars co‐exist in environments but its regulatory network and functions as a regulator of carbon metabolisms and photosynthesis remain unknown (Oesterhelt & Gross, 2002). *C. merolae* genome also encodes two hexokinases (CMS263C and CMO276C). It is worth investigating these proteins in GsSPT1‐expressing cells will give insights into how photosynthetic carbon fixation and glucose flux are regulated in *C. merolae*.

### 4.2 Possibilities for further improvement of *C. merolae* heterotrophic culture

It took about 2 weeks to obtain visible colonies of Cp‐*SPT1* cells in the light on a solidified medium with DCMU and glucose (Figure 4c). This duration is within practical use because it takes 1–4 weeks to obtain colonies of photosynthesis‐deficient *C. reinhardtii* and *Synechocystis* sp. PCC6803 mutants (Przibilla, Heiss, Johanningmeier, & Trebst, 1991; Spreitzer & Mets, 1981; Yamasato & Satoh, 2001). However, in *C. reinhardtii*, many photosynthesis‐deficient mutants are light‐sensitive (ie, grows slower in the light than dark in the presence of acetate; Spreitzer & Mets, 1981).

When a photosynthesis‐deficient *C. merolae* mutant exhibits a similar light‐sensitive phenotype, periodic light pulsing is likely effective to improve heterotrophic culture conditions according to the following finding. In *C. merolae*, a daily light pulse (10 min of 30 μmol m^−2^ s^−1^) was required for continual heterotrophic growth with 200–400 mM glycerol (Moriyama et al., 2018). Such light‐activated heterotrophic growth was also reported in *Synechocystis* sp. PCC6803 (Anderson & McIntosh, 1991). In *Synechocystis* sp., it is assumed that light pulse likely functions as an environmental signal to regulate heterotrophic metabolisms, cell division, and other cellular activities (Anderson & McIntosh, 1991).

## CONFLICT OF INTEREST

The authors declare no conflict of interest associated with the work described in this manuscript.

## AUTHOR CONTRIBUTION

TF and S‐YM conceived and designed the experiments and also wrote the paper. TF, YK, MM, and SW performed the experiments. TF, HS, RO analyzed the data. TF contributed the reagents/materials/analysis tools.
