# Supplementary figures and images for: Integration of a Galdieria plasma membrane sugar transporter enables heterotrophic growth of the obligate photoautotrophic red alga Cynanidioschyzon merolae
Source: Plant Direct. 2019 Apr 8;3(4):e00134. doi: 10.1002/pld3.134 (PMC6589524; doi:10.1002/pld3.134)

# Supporting Information FIGURE S1

(a)

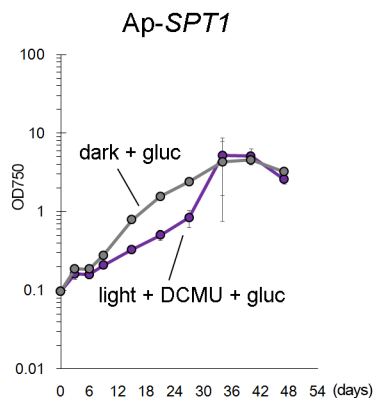

(b)

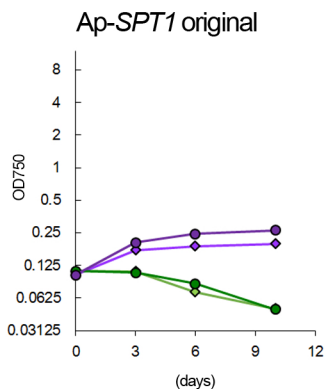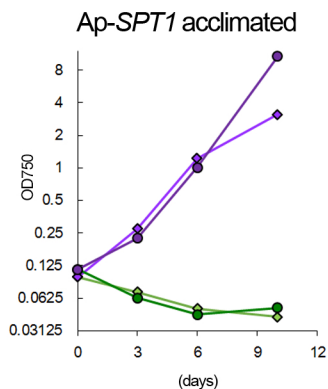

- ◆ #1 light + DCMU
- ◆ #1 light + DCMU + Gluc
- #2 light + DCMU
- #2 light + DCMU + Gluc

Supplement: Supplementary file 1 [file PLD3-3-e00134-s001.pdf]
