# Supplementary material for: Integration of a Galdieria plasma membrane sugar transporter enables heterotrophic growth of the obligate photoautotrophic red alga Cynanidioschyzon merolae
Source: Plant Direct. 2019 Apr 8;3(4):e00134. doi: 10.1002/pld3.134 (PMC6589524; doi:10.1002/pld3.134)
Supplement: Supplementary file 2 [file PLD3-3-e00134-s002.pdf]

Supporting Information TABLE S1. The list of primers used in this study

No.	Primers for plasmid construction	Sequences
1	APCC(-1)R	GGTCAACGAACGAAGAAACACAG
2	bTub3'(+1)_1	TAAACTAGCTATTTATCTGGTACATATCATTCAT
3	HA(1)Fapcc	cttcgttcgttgaccATGTACCCATACGATGTTCCTGAC
4	HA(90)R	AGCGTAATCTGGAACGTCATAAGG
5	GsSPT1(4)Fha	gttccagattacgctGAGGCTATAACCGAATTCCA
6	GsSPT1(1671)Rbt	taaatagctagtttaTTTAGAAACTTCACCAGCAT
7	URA(-2300)F	CTTCAAGAAAAGAGGATCTTTTGCCGTGATGCC
8	URA(+471)R	CCCTAGCAGCTGACTGTATCTCTATTCTTAGGAAT
9	D184(+25)R	CGTCACCCTCGGGACTTGATGTTTACGTTC
10	btub(+1)F	TAAACTAGCTATTTATCTGGTACATATCATTCATAAGCACATGTTTTTG
11	CPCC(-500)Fd184	gtcccgagggtgacgCACTAGTCCGGTGAACTCGG
12	CPCC(-1)R	ATCAGAAGTGCTTTACGAGGAAC
13	D184(1200)F	CGCCTTCTCCTGGACGAGTAC
14	D184(+1400)R	CCAGAGCCCTACCGGCA
15	URA(-898)R	AGTCATACAACAGTACTCAGATCGTTGAGGAACAATGAAAG
16	mVENUS(1)Fcpcc	taaagcacttctgatATGGTTAGCAAGGGCGAAGAGC
17	CPCC(-500)Fura898	tactgttgtatgactCACTAGTCCGGTGAACTCGG
18	CPCC(-1)R	atcagaagtgctttacgaggaacgt
	**Primers for qRT-PCR**	**Sequences**
19	btub(+108)F	TCGACCAGCTCTCGAACAAA
20	btub(+169)R	TCTGGTCAATACCGCAGCAA
21	URA(-500)F	GGGCGAGGTAGGTGCTAGTTT
22	URA(-439)R	GTATTCCGCAGCCGTTGTG
23	URA(+473)F	TTGTGGCGCGACTTTCG
24	URA(+525)R	GCTGTACCCCGATTCGATCA

Lowercase letters indicate adaptor sequences for In-Fusion reaction.
