# Supplementary material for: Integration of a Galdieria plasma membrane sugar transporter enables heterotrophic growth of the obligate photoautotrophic red alga Cynanidioschyzon merolae
Source: Plant Direct. 2019 Apr 8;3(4):e00134. doi: 10.1002/pld3.134 (PMC6589524; doi:10.1002/pld3.134)
Supplement: Supplementary file 3 [file PLD3-3-e00134-s003.pdf]

Supporting Information MATERIALS S1. The nucleotide sequences of *mVENUS* and HA-*SPT1* genes used in this study.

>mVENUS (codon-optimized mVENUS)
(*APCC5*'cttcgttcgttgacc-)ATGGTTAGCAAGGGCGAAGAGCTGTTTACGGGCGTGGTCCCAAT
CCTGGTGGAACTCGACGGCGATGTCAACGGTCACAAATTCTCGGTGTCGGGCGAGGGCG
AGGGTGATGCAACCTACGGCAAGCTGACGCTCAAGCTGATCTGCACGACCGGCAAGCTG
CCAGTGCCATGGCCGACCCTGGTCACCACCCTGGGTTACGGCCTCCAGTGCTTCGCACG
CTACCCGGACCACATGAAGCAGCATGATTTCTTTAAGAGCGCAATGCCAGAGGGTTACGT
GCAGGAGCGCACGATCTTCTTTAAGGACGATGGCAACTACAAGACGCGCGCCGAGGTGA
AGTTCGAGGGCGACACGCTGGTCAACCGCATCGAGCTCAAGGGCATCGACTTTAAAGAG
GACGGCAACATCCTGGGCCACAAGCTCGAGTACAACTACAACAGCCATAACGTCTACAT
CACGGCGGATAAGCAGAAGAACGGCATCAAGGCCAACTTTAAGATCCGCCACAACATCG
AGGATGGCGGCGTGCAGCTGGCAGATCATTACCAGCAGAACACCCCAATCGGTGATGGT
CCAGTCCTGCTCCCGGATAACCACTACCTGTCGTACCAGAGCGCACTCTCGAAGGACCC
GAACGAGAAGCGCGATCATATGGTGCTCCTGGAATTTGTGACGGCGGCGGGCATTACGCT
GGGTATGGATGAACTGTATAAG(-taaactagctattta, *β-tub* ter)

≥HA-*SPT1* (codon-optimized triple HA-tag fused *SPT1*)
(*CPCC5*'taaagcacttctgat-)ATGTATCCTTATGACGTCCCGGACTATGCCGGCTACCCATATGATG
TGCCGGACTATGCTGGATACCCGTATGACGTACCCGACTATGCGGAGGCCATCACTGAGT
TCCAGCAAGCGGCTGAGCCCAAAAATGACTTGTACGATTCCACCGAGCTTCAAAAAGAA
GCGGGGGTGACGGACCTGGATGATGAGTACCTCACTGAAGAGGAGCGTCTGCACCCTTG
GCGTCAAACGCGCACCTTCTATTGGAGCGTGATTGTGGCGAGCGTTGCTGTCCTGTTTTA
TGGCATCGATATCACCGCCATTTCGGGCGCGCAGGTGGGCTACATACCCCATTTCGGCCTG
GAAAATCGCACGTACCACCAGGGCGTCATGGTGAGCTGTATCTATTTCGGTTGCTTCGTA
GGAGTGTTTATCGCTCTCTTCACGAATACCTATTTCGGCCGCCGCTTCACGATCAGCCTGG
CCTCGTTCATTTCCACGGGAGCGTGTATATGGGAGGCGGTGTCGCCATCGTGGCAAGTCT
TCATCCCCGGACGTATCATCCTGGGCTTTTCGTACGGAATGATCGGTGAGACTGCGCCGG
TCTATCTGGCGGAAATGTCCCCGGCGAGCATTCGTGGCGCCATTGTGAGCCTCTACCAGC
AGGTGGTGACTATCGGCATATTCCTCGCGTACCTGTGCAATCTGATATTCGTCTGGGTCAA
CTACCAAAACGTCGGATGGCGCATCATGATAGGTTTCCCGATGGTCCCCTCGGTGATCGA
GATGTTTCTGATCTGGACGGTTCCGGAGAGCCCCCGCTGGCTGATTAAACGCCGCCGCTA
CGAGGATGCTAAACGGAACCTTTTCAAGCTCCGGCGCACCGCGGAGACCGCGGAGCGC
GATTTCGTCCGGATCAAGAAAGGCGTTGAAGAGGATGAGATCCTGCAAAAAGGTAAGAA
TCTGCTGGTAGAGGTGATCCCTGTTCCTTACATTCGACGCGCACTCCTGATTGGAATCATG

GAAATGCTGTTCCAGCAGATGTCGGGCATGAACGTCTTTATGAATTACATCGACGAAGTG
TTCGAGGAGAACATCAACATGGGGGCCCGCACATCAGTCGCGGTCAGTCTCTTCCCAGG
CTTCGTAAACATGGTGGCAACGGTCATCGTATACTTCACCATCGATCGGTACGGTCGTCG
GACTTTGCAGCTGGTCACCTTCCCTGTAATGTTTTTGATGCTTCTCATGGTGCTTTTCTCG
TTTTACGGCGACAAGAAAGTTAACCTCGCTTTTTTCATCATTGGTGTGGTGTTTTTTATTG
TGGCCTACAGTCCCGGAGCCGGGCCAGTTCCGTGGACCTTTTGCGCGGAAGTGTTTCCA
ACATACGTTCGGGCAGCCGGCACGACGATTACAACGTTCTTCGTTAACGCTTTTAACTTC
GCACTCAGTTTCTCCTGGCCATCCATGAAGGCCGCTTGGGGCCCGCAGGGCGGTTTTGGT
TTTTACGCCGGGTTTAACTTCCTCGGGATTGTTATGCAGTTTTTGTTTCTTCCGGAAACGA
AGGGTTTTACATTGGAACAGATGCGTGTTGTTTTTGAAGAGGGTCTCTTTACAATTGCAG
CATACCATTGCCGAGCAGGGTGGCGGAGTTTGCGAAAGCTCTTGGGTCTTTCAGTTCCTG
ACACACCGCTTGTTTCACCATACGACAAGGCATTTGCAATTGACCGAGCAAAGCGAGAG
GAAGAAATGATGCACGCAGGTGAAGTTTCAAAG(-taaactagctattta, *β-tub* ter)

The triple HA tag sequence is underlined. Lowercase letters indicate adaptor sequences for In-Fusion reaction.
