# Supplementary material for: Integration of a Galdieria plasma membrane sugar transporter enables heterotrophic growth of the obligate photoautotrophic red alga Cynanidioschyzon merolae
Source: Plant Direct. 2019 Apr 8;3(4):e00134. doi: 10.1002/pld3.134 (PMC6589524; doi:10.1002/pld3.134)
Supplement: Supplementary file 4 [file PLD3-3-e00134-s004.pdf]

Dear Dr. Farre, and Dr. Fortes,

We thank you and the reviewer for the helpful and important comments to improve our manuscript (2019-00257). We have considered all of the comments carefully and made the following revisions, which are highlighted in the uploaded text file. The reviewer's comments were copied and underlined and we have described our revisions directly beneath each comment.

Reviewer #1:
In this manuscript, Fujiwara et al. generated a transgenetic line of C. merolae and achieved heterotrophic and mixotrophic growth of this alga. This research provides an explanation for the inability of wild-type C. merolae to grown heterotrophically, in contrast to some related red algal species. It also establishes a new system for genetic identification of photosynthesis in the red lineage. The manuscript is generally well-written. To improve the manuscript, I suggest the following changes:

Thank you very much for your positive evaluation on our manuscript. We have revised the manuscript according to your suggestion as describe below.

Major comments:
1. Because G. sulphuraria already allows generation of photosynthesis-deficient mutants in red algae, the authors should explain clearly the unique utility for heterotrophically-growing C. merolae to be used for photosynthesis research.

Thank you for your suggestion. We have added following statements in the text (pages 7-8, lines 130-135).

Thus, it is probably feasible to obtain photosynthesis-deficient mutants of *G. sulphlaria* by random mutagenesis. However, further molecular genetic analyses will be limited because G. sulphuraria is not genetically tractable. In addition, a rigid cell wall of *G. sulphlaria* hampers extraction and fractionation of cellular contents in mild processing conditions. This situation contrasts with *C. merolae* which does not possess a rigid cell wall.

Minor comments:

2. Lines 763-764, format error.

We have fixed the error.

Reviewer #2:

In this paper, the authors propose a novel tool for studying photosynthesis in *C. merolae*, by use of a sugar transporter from *Galderia* to enable heterotrophic or mixotrophic growth. Their approach has merit and is generally well-documented. They comprehensively characterize and demonstrate heterotrophic growth by combinations of light/dark treatments, DCMU treatments and glucose titrations in SPT1 expressors and WT. There are a few concerns which, when addressed, would improve the quality of the manuscript.

Thank you very much for your positive evaluation on our manuscript. We have revised the manuscript according to your suggestion as describe below.

Major Comments

1. Line 386: As a component of the phycobilisomes does this promoter show light activation? Could a light activation of the promoter explain the improved growth in the light of the transgenics?

Thank you for your important suggestion. We have newly added an immunoblotting result showing SPT1 level (expressed by *CPCC* promoter) in the culture in the dark with glucose and that in the light with glucose and DCMU (Fig. 4b). A previous paper showed that *CPCC* mRNA level in the dark was lower than that in the light under photoautotrophic condition (Kawase et al., 2017). However, in our result in heterotrophic conditions (Fig. 4b), SPT1 protein level was not so much different between CP-*SPT1* in the dark with glucose and that in the light with glucose and DCMU. Therefore, the difference in the growth will be because of reasons other than the light response of *CPCC* promoter. We have added the description of the result and discussion in the text (Page 22, lines 468-475).

2. Making *C. merolae* hetero or mixotrophic could indeed help advance studies of photosynthesis in this unique red algae, but there should be some comment on the altered physiology resulting from direct sugar import. For example, what is known

about sugar sensing in C. merolae or related species?

Thank you very much for your comment. We have added following statements in the text (Page 26, lines 564-576).

Regarding the physiological changes resulting from direct sugar uptake in GsSPT1-expressing cells and the mechanisms regulating a metabolic balance between photosynthetic carbon fixation and glucose flux, further studies are required. Hexokinases are known as evolutionarily conserved sugar sensors in eukaryotes and regulate expressions of genes involved in sugar uptake, carbon metabolism and photosynthesis (Rolland et al., 2006; Aguilera-Alvarado and Sa´nchez-Nieto 2017). In *G. sulphuraria*, it is assumed that hexokinases are involved in selection of a preferred sugar to be taken up when various kinds of sugars co-exist in environments but its regulatory network and functions as a regulator of carbon metabolisms and photosynthesis remain unknown (Oesterhelt and Gross, 2002). *C. merolae* genome also encodes two hexokinases (CMS263C and CMO276C). It is worth investigating these proteins in GsSPT1-expressing cells will give insights into how photosynthetic carbon fixation and glucose flux are regulated in *C. merolae*.

3. Many of the conclusions of this work assume the DCMU treatment completely blocked linear electron transport. It was not clear what concentrations where used for which experiments and how it was confirmed that the treatments blocked linear electron flux.

Thank you for pointing out an important point. The concentration of DCMU was 10 µM throughout the experiments where used and these are indicated in Figs. 2c and 4a and respective figure legends (Page 38, lines 833-835).

We have compared oxygen evolution rates in the light/dark with or without 10 µM DCMU (Fig. 2c; the method has been described on pages 13-14, lines 250-229). The result showed that 10 µM DCMU completely abolished linear electron flow in the photosystems (Page 17, lines 354-361).

Minor Comments

4. Line 76: Is there a more updated review for the use of cyanobacteria as a model organism?

We have added Peschek et al., 2011 (Page 5, line 75).

5. Line 130: Given the ability of G. sulphuraria to grow heterotrophically and its similarity to C. merolae, why not just use it as a model organism instead of C. merolae?

Thank you very much for pointing it out. We have added following statements (Page 7-8, lines 130-135).

Thus, it is probably feasible to obtain photosynthesis-deficient mutants of *G. sulphlaria* by random mutagenesis. However, further molecular genetic analyses will be limited because *G. sulphuraria* is not genetically tractable. In addition, a rigid cell wall of *G. sulphlaria* hampers extraction and fractionation of cellular contents in mild processing conditions. This situation contrasts with *C. merolae* which does not possess a rigid cell wall.

6. Line 164: At what temperature were the cultures maintained? What was the pH and how often was it checked?

Thank you for pointing it out. We have added information in the Materials and Methods as follows (Page 9, lines 167-168 and lines 173-175).

The wild-type *C. merolae* 10D (NIES-3377) and the *C. merolae* M4-derived transformants were maintained in inorganic MA2 medium, which was adjusted to pH 2.3 with H_2SO_4 (Ohnuma et al., 2008), in 60 mL tissue culture flasks (90026, TPP) under continuous light (photon flux 50 µmol m^{-2} s^{-1}) at 42 ˚C.

The pH of cultures in several different conditions was checked 10 days after inoculation. The pH values were between 2.2 to 2.4. These were within the range for the optimal growth of *C. merolae* (pH 1.0 - 4.0; Kuroiwa *et al*., 2017).

7. Line 255: It would be better to list molar concentrations of primers and DNA from the qPCR experiments

We have added the information in the Materials and Methods as follows (Page 14, lines 269-270).

Quantitative PCR was performed using the StepOnePlus Real-Time PCR System (Applied Biosystems) with a 20-μL reaction mixture [10 μL of PowerUp SYBR Green Master Mix (Thermo Fisher Scientific), 0.1 μL of 50 μM forward and reverse primers, 4 μL of each 0.5 ng/μL genomic DNA solution, and 15.8 μL of water]. The primers used in this study are listed in Supporting Information Table 1.

8. Line 327: Could the similar initial growth rates and higher final growth level be due to exhausting the glucose in the media? Could this also indicate transport limitation? It should be noted how the transformed lines will be made available to other researchers.

Thank you very much for pointing it out. We have added following statements (Page 16, lines 340-344).

The similar initial growth rate among different concentrations of exogenous glucose was probably because of limitation of glucose uptake activity of the HA-SPT1 proteins. In addition, the difference in their final cellular concentrations was probably because of exhaustion of exogenous glucose.

We have added following statement in Acknowledgements (Page 27, lines 601-602).

The strains described in this manuscript are available from TK upon request.

9, Line 346: This is an interesting observation since this is about the same time that growth levels off in Ap-SPT1 under 25 mM glucose and no DCMU.

Yes, the growth of Ap-*SPT1* cells with DCMU and glucose was suddenly accelerated from day 29 to 41 until exogenous glucose was exhausted. The final cellular concentration and the duration for reaching the concentration were almost the same

between the culture in the dark with glucose and that in the light with glucose and DCMU (Fig. 2b). However, the reasons remain unclear at this point.

10. Line 469: Why is it considered a putative sugar transporter here, but seems more confirmed in other parts of the paper.

Thank you for your comment. We agree with the comment. We have removed “putative” because the sugar uptake ability of GsSPT1 was already shown in a previous study (Schilling and Oesterelt 2007).

11. Line 526: The wording in this sentence could be improved. Perhaps "kept" could be removed.

We have removed “kept” according to your suggestion.

12. Line 547 to the end of the paragraph: The wording of this section could be improved.

We have modified that part as followings (Pages 26-27, lines 586-594).

When a photosynthesis-deficient *C. merolae* mutant exhibits a similar light-sensitive phenotype, periodic light pulsing is likely effective to improve heterotrophic culture conditions according to the following finding. In *C. merolae*, a daily light pulse (10 min of 30 µmol m^{-2} s^{-1}) was required for continual heterotrophic growth with 200-400 mM glycerol (Moriyama et al., 2018). Such light-activated heterotrophic growth was also reported in *Synechocystis* sp. PCC6803 (Anderson and Mcintosh, 1991). In *Synechocystis* sp., it is assumed that light pulse likely functions as an environmental signal to regulate heterotrophic metabolisms, cell division, and other cellular activities (Anderson and Mcintosh, 1991).

We believe our manuscript has been improved, thanks to the referees, and hope it is now acceptable for publication in *Plant Direct*. We look forward to your decision.

Yours sincerely,

Takayuki Fujiwara
Shin-ya Miyagishima
